# Radiation-induced liver injury and hepatocyte senescence

**DOI:** 10.1038/s41420-021-00634-6

**Published:** 2021-09-16

**Authors:** Wei Zhu, Xiaofen Zhang, Mengli Yu, Bingru Lin, Chaohui Yu

**Affiliations:** Department of Gastroenterology, First Affiliated Hospital, School of Medicine, Zhejiang University, Hangzhou, China

**Keywords:** Senescence, Mechanisms of disease

## Abstract

Radiation-induced liver injury (RILI) is a major complication of radiotherapy during treatment for liver cancer and other upper abdominal malignant tumors that has poor pharmacological therapeutic options. A series of pathological changes can be induced by radiation. However, the underlying mechanism of RILI remains unclear. Radiation can induce cell damage via direct energy deposition or reactive free radical generation. Cellular senescence can be observed due to the DNA damage response (DDR) caused by radiation. The senescence-associated secretory phenotype (SASP) secreted from senescent cells can cause chronic inflammation and aggravate liver dysfunction for a long time. Oxidative stress further activates the signaling pathway of the inflammatory response and affects cellular metabolism. miRNAs clearly have differential expression after radiation treatment and take part in RILI development. This review aims to systematically profile the overall mechanism of RILI and the effects of radiation on hepatocyte senescence, laying foundations for the development of new therapies.

## Facts

- Normal tissue receiving radiation during radiotherapy or radioscopy will suffer injury and metabolic alterations.
- Reactive oxygen species (ROS) directly and indirectly generated by radiation play an important role in liver injury initiation and aggravation.
- Both hepatocytes and nonparenchymal cells are involved in RILI progression via the activation of several signaling pathways.

## Questions

- Why do ROS-targeted treatments exhibit controversial effects? Is this result related to the state of the oxidation/reduction system and timing after radiation?
- RILI is a complex process involving a variety of cells and signals; can it be phased according to cellular biological processes to find the best treatment method?
- The DNA damage response contributes to hepatocyte senescence during RILI, so what are its differences and common points compared with natural aging?

## Radiation

Radiation is defined as the transmission or emission of energy in the form of waves or particles. It can immediately generate highly reactive free radicals, resulting in rapid protein modifications and damage to DNA, RNA, and cell membranes [1, 2]. The molecular events after radiation are complicated and span a variety of biological processes [1], including senescence [3–5], oxidative stress [6], inflammation, the depletion of injured cells, and fibrosis [7].

The liver is a very important organ that participates in various physiological functions, such as bile production, lipid metabolism, glycometabolism, elimination of various waste products, immunity, and plasma protein synthesis. While exposed to radiation due to a nuclear accident or as an intended treatment for cancer, as a radiosensitive organ, the liver may suffer from radiation-induced liver injury (RILI), resulting in hepatitis, fibrosis, cirrhosis, and cancer. Typical pathological appearances of RILI in humans are perivenular fibrosis, sinusoidal obstruction, and damage to Kupffer cells (KCs) and hepatocytes [8]. The severity of RILI depends upon the nature of the radiation, the total exposure dose, the dose rate, and the physical area of exposure [3].

RILI occurring in advanced liver cancer during radiotherapy, particularly for a cirrhotic liver, can be potentially life-threatening. Clinical practice to modify radiation parameters and prevent RILI have been well described and applied during medical activities [9, 10]; however, no pharmacological therapies have demonstrated adequate effects to alleviate RILI once it has manifested clinically [11]. Here, we aim to discuss the mechanism of RILI and the effects of radiation on hepatocyte senescence, laying foundations for the development of new pharmacological therapies.

## Radiation and senescence

Senescence is a multistep, dynamic cellular process. Senescence-inducing signals, such as oncogene activation, DNA damage, and telomere shortening (replicative aging), can induce cell cycle arrest and/or senescence initiation [12]. Senescent cells no longer proliferate but remain metabolically active for a long time. Cellular senescence is mainly characterized by a combination of multiple markers, such as morphological and metabolic changes, expression of cell cycle inhibitors, senescence-associated-β-galactosidase (SA-β-gal) activity, SASP activity, and changes in the nuclear membrane [13].

Cellular senescence is considered a complication of radiation following the activation of the DDR. This response provides cells with the ability to sense and signal damage in its DNA, arrest cell cycle progression (cell cycle checkpoints) and activate appropriate DNA repair mechanisms, or eliminate cells with unrepairable genomes [14]. When DNA damage is not successfully repaired, it can result in senescence induction as a functional alternative to apoptosis. DNA double-strand breaks (DSBs) are considered to be the most serious type of DNA damage induced by radiation [15, 16]. Key drivers of the DDR include ataxia telangiectasia mutated (ATM), ataxia telangiectasia and Rad3-related (ATR), and the DNA-dependent protein kinase catalytic subunit (DNA-PKcs) [16]. Among them, ATM and DNA-PKcs are mainly activated by DSBs [17]. Their phosphorylated substrates have important roles in the functions of cell cycle checkpoints and cell death, as well as in DSB repair [18]. For example, it has been reported that ATM can be activated by radiation through intermolecular autophosphorylation and dimer dissociation [19]. Activated ATM kinase can phosphorylate several proteins, such as p53, MDM2 and CHK2 in the G1 checkpoint; NBS1, BRCA1, FANCD2 and SMC1 in transient radiation-induced S-phase arrest; and Brca1 and hRad17 in the G2/M checkpoint [19]. These substrates participate in radiation-induced cell cycle arrest.

Le et al. [4] confirmed that after exposure to a sublethal dose of radiation, the liver senescence markers P53-binding protein 1 (53BP1) and p16 peaked after a short period of time, then gradually decreased, persisting for as long as 45 weeks. Damaged cells were preferentially eliminated; however, a high level of senescent markers remained compared with normal tissue. Furthermore, experiments in p53 −/− mice and Rag2 −/− γC −/− mice verified that the accumulation of senescent cells was independent of p53 and an intact immune system.

The study by Serra et al. [20] showed that similar to 40% liver mass hepatectomy, a single dose of 25 Gy radiation could induce hepatocyte senescence in rats. Several markers of cell senescence were upregulated in hepatocytes receiving radiation, including the expression of SA-β-gal, an increase in cell size, upregulation of p16 and p21, and activation of SASPs, such as IL6 and IL1α.

SASP secreted from senescent hepatocytes is regarded as the main medium leading to changes in tissue homeostasis and microenvironment. The expression and secretion of many proinflammatory cytokines, chemokines, growth factors, and proteases in senescent cells are termed the SASP. SASP can reinforce senescence growth arrest in an autocrine manner or promote the conversion of nonsenescent cells to senescent cells in a paracrine manner [12, 13]. It may also directly or indirectly promote chronic inflammation linked to metabolic dysregulation, stem cell dysfunction, chronic diseases, and tumors [21].

In two different fibroblast cell strains, Marthandan et al. [22] compared the corresponding transcriptional differences between replicative and radiation-induced senescence. Despite the different senescence-inducing signals, there was a high degree of similarity in the differential gene expression, mainly involved in cell cycle regulation. Additionally, compared to replicative senescence, they found that the pathways associated with “DNA repair” and “replication” were less stringently regulated in radiation-induced senescence. Similarly, Aliper et al. [23] demonstrated a significant concordance between radiation-induced and replicative-induced senescence signals in fibroblasts. Additionally, they found that the transcriptome of replicative senescent fibroblasts was more similar to the transcriptome of cells receiving 2 Gy of radiation than those receiving 5 cGy. In addition, the study by Casella et al. [24] confirmed that senescence profiles were more dependent on the cell of origin than on the method of induction. Accordingly, we supposed that for a particular type of cell exposed to radiation, cell fate depends on the radiation dose, while the senescent signal activated by radiation is highly conserved compared with natural aging.

In general, radiation can induce cellular senescence via activation of the DDR. Hepatic senescent cells accumulate after radiation. The enlarged proportion of senescent cells results in the loss of the regenerative and homeostatic capacities of the liver. Furthermore, this creates a persistent proinflammatory microenvironment, which plays an important role in the process of fibrosis and tumorigenesis and aggravates the development of RILI. Senolytic agent small molecules can selectively kill senescent cells. The use of senolytic agents has been approved to improve organ function after radiation-induced organ injury. ABT-737, a small molecule inhibitor targeting the BCL-2, BCL-W and BCL-XL proteins, can eliminate radiation-induced senescent cells in the lung mediated by an increase in apoptosis [25]. In addition, another senolytic agent, ABT-263, can selectively kill radiation-induced senescent hematopoietic stem cells, promote the expansion of normal hematopoietic stem cells, and abrogate radiation-induced SASP secretion [26]. Treatment of radiated mice with ABT-263 reduced senescent cell numbers and restored a regenerative phenotype in the kidneys with increased tubular proliferation and improved function [27]. Although there are currently no relevant studies on RILI, senolytic agents are beneficial in part by their ability to rejuvenate injured organs and may represent a new method to ameliorate RILI.

## Oxidative stress

**Fig. 1:**
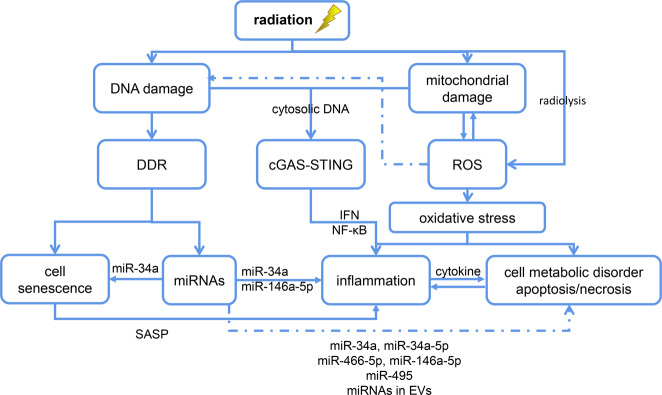
Various biological processes are involved in RILI. After radiation exposure, excessive production of ROS in cells leads to oxidative stress and inflammation, resulting in cell damage. DNA damage in the nucleus and mitochondria can aggravate cell damage by activating cGAS-STING signal pathway. Cellular DNA damage response can participate in the occurrence and development of RILI by regulating the expression of specific miRNA and inducing cell senescence. DDR: DNA damage response; ROS: Reactive oxygen species; SASP: senescence-associated secretory phenotype; EVs: extracellular vesicles.

Radiation energy can result in radiolysis of water in cells and tissues, which induces the immediate production of ROS and reactive nitrogen species (RNS) [15, 28]. A few hours after exposure, the oxidation/reduction system begins to produce free radicals after the direct cellular damage that is caused by radiation energy [6]. Eventually, cell and tissue damage occurs secondary to the activation of a series of biological pathways (Fig. 1).

After exposure to radiation in the whole body or liver region, cellular components such as proteins [3, 29], lipids [30, 31], and nucleic acids [32, 33] undergo oxidative stress.

Protein carbonylation [3] and nitration [29, 34] are commonly used markers of oxidative stress in liver proteins. As described by Barshishat-Kupper et al. [3], total carbonylation increased after radiation, reaching a peak 48 h after radiation. In addition, they discovered that the carbonylation level of carbonic anhydrase 1, a-enolase, and regucalcin specifically increased, which is associated with metabolic alterations in hepatic functions [3]. Cumulative protein carbonylation has been demonstrated to impair protein structure and function [35] due to the inability to degrade these extensively oxidized proteins, as well as protein nitration [36].

The lipid peroxidation level is estimated by measuring the levels of lipid peroxidation (LPO) [37], methylenedioxyamphetamine (MDA), 4-hydroxynonenal (4-HNE) [30], and thiobarbituric acid reactive substance (TBARS) [31]. Membrane lipid peroxidation can enhance the rigidity of membranes, decrease the activity of membrane-bound enzymes, change membrane receptor activity, and alter membrane permeability [15].

Numerous lesions can occur in DNA following radiation exposure, including oxidized bases, the loss of bases, DNA-DNA intrastrand adducts, DNA–DNA and DNA-protein crosslinks, single-strand breaks (SSBs) and DSBs [15]. 53BP1 and γ-H2AX, which are produced after DDR activation, are usually used as DNA damage markers [4]. The common marker of DNA oxidation is 8-hydroxy-deoxyguanosine (8-OHdG) [32].

Additionally, the activities of superoxide dismutase (SOD) [38], catalase (CAT) and GSH transferase (GSH-T), the content of reduced glutathione (GSH), and the ferric reducing antioxidant power (FRAP) are diminished upon radiation exposure compared with controls, while the activity of the detoxification enzyme cytochrome P450 (CYP450) increases [31, 32].

The increased expression of the markers mentioned above can be observed hours after radiation and last for several weeks or even months (Table 1).

**Table 1:** RILI markers observed after radiation exposure.

RILI markers	Response	Observed time	Type of radiation	Dose	Rf
ALT	Increase	1d, 7d, 10d, 14d, 15d	X-ray, γ-ray	5 Gy,6 Gy,6.5 Gy,7 Gy,9 Gy,10 Gy,15 Gy,30 Gy	[30, 32, 33, 37–39, 55, 100, 101]
AST	Increase	1d, 7d, 10d, 14d, 15d	X-ray, γ-ray	5 Gy,6 Gy,6.5 Gy,7 Gy,9 Gy,10 Gy,15 Gy,30 Gy	[30, 32, 33, 37–39, 55, 100, 101]
ALP	Increase	1d, 3d, 5d, 10d, 15d	X-ray,γ-ray	6 Gy,9 Gy,15 Gy,30 Gy	[30, 32, 39, 100]
GGT	Increase	1d, 14d, 15d	γ-ray	6 Gy,9 Gy,10 Gy	[32, 38, 100]
ROS	Increase	10d	X-ray	15 Gy	[38, 55, 100, 101]
CAT	Decrease	6 h, 1d, 2d, 7d, 15d	X-ray, γ-ray	5 Gy,6 Gy,6.5 Gy,7 Gy,9 Gy	[31, 32, 37, 55, 100–102]
CYP 450	Increase	1d, 7d	γ-ray	5 Gy,7 Gy,9 Gy	[32, 54, 101]
GSH	Decrease	6 h, 1d, 2d, 7d, 15d	γ-ray	5 Gy,6 Gy,6.5 Gy,7 Gy,9 Gy	[31, 32, 37, 55, 100–102]
GSH-T	Decrease	1d, 7d	γ-ray	7 Gy,9 Gy	[32, 101]
SOD	Decrease	1d,7d, 14d, 15d	γ-ray	5 Gy,6 Gy,7 Gy,10 Gy	[30]
FRAP	Decrease	6 h	γ-ray	5 Gy	[31]
Protein carbonylation	Increase	1d	X-ray, γ-ray	4 Gy,7 Gy,9 Gy	[32, 103]
LPO	Increase	7d	γ-ray	6.5 Gy	[37]
4-HNE	Increase	10d	X-ray	15 Gy	[30]
MDA	Increase	1d, 2d,7d, 10d, 14d, 15d	X-ray, γ-ray	4 Gy,6 Gy,7 Gy,9 Gy,10 Gy,15 Gy	[30, 32, 38, 100–103]
TBARS	Increase	6 h, 1d	γ-ray	5 Gy	[31, 55]
8-OHdG	Increase	1–7d	X-ray,γ-ray	9 Gy,30 Gy	[32, 33]
53BP1	Increase	1d, 1w, 4w, 12w, 21w, 45w	X-ray	8 Gy	[4]

*ALT* alanine transaminase, *AST* aspartate transaminase, *ALP* alkaline phosphatase, *GGT* gamma-glutamyltransferase, *ROS* reactive oxygen species, *CAT* catalase, *CYP 450* cytochrome P450, *GSH* reduced glutathione, *GSH-T* GSH transferase, *SOD* superoxide dismutase, *FRAP* ferric reducing antioxidant power, *LPO* lipid peroxidation, *4-HNE* 4-hydroxynonenal, *MDA* methylenedioxyamphetamine, *TBRAS* thiobarbituric acid reactive substance, *8-OHdG* 8-hydroxy-deoxyguanosine, *53BP1* p53-binding protein 1

Numerous studies have revealed that antioxidants can alleviate the progression of RILI in both the short term and long term by downregulating inflammatory reactions and apoptosis (Table 2) [29–32, 37].

**Table 2:** Antioxidants alleviate the progression of RILI.

Antioxidants	Natural or synthetic	Mechanism	RILI models	Type of radiation	Dose	Ref
Salen Mn complex (EUK-189 and EUK-207) and Mn porphyrins	Synthetic	Novel Synthetic SOD/Catalase Mimetics possess superoxide dismutase (SOD), catalase and peroxidase activities.	Capillary endothelial cells	X-ray	2-50 Gy	[28]
GC4401	Synthetic	Highly specific superoxide dismutase mimic	Mice	γ-ray	4 Gy	[29]
P. ginseng water extract	Natural	/	Mice	X-ray	15 Gy	[30]
Epicatechin	Natural	/	Mice	γ-ray	5 Gy	[31]
Betaine	Natural	/	Rats	γ-ray	9 Gy	[32]
Date syrup	Natural	/	Rats	γ-ray	6 Gy	[102]
Persimmon leaf	Natural	/	Rats	γ-ray	6 Gy	[100]
Grape seed oil	Natural	/	Rats	γ-ray	7 Gy	[101]
Flaxseed oil	Natural	/	Mice	γ-ray	5 Gy	[96]
Astragalus polysaccharide	Natural	/	Mice	γ-ray	5 Gy	[55]

The study by Coleman et al. [29] confirmed that GC44401, a highly specific superoxide dismutase mimic, could prevent superoxide anion (•O_2_−)-mediated acute liver injury in SIRT3 −/− mice exposed to whole-body γ-radiation. Another study pointed out that in SIRT3 −/− mice, the activation of hydrogen peroxide- and hydroperoxide-sensitive signaling cascades was involved in long-term RILI [34]. However, Liu et al. [39] revealed that glibenclamide elevated the cell membrane potential to upregulate intracellular ROS, which subsequently activated the active (Akt)-nuclear factor kappa-B (NF-κB) pathway to promote the survival of radiated hepatocytes. The application of N-acetylcysteine (NAC), a specific ROS scavenger, eliminated the protective effects of glibenclamide [39].

It seems that ROS is a double-edged sword in the development of RILI, and its role is not fully understood.

## Glucose and lipid metabolism disorders

The liver is the main organ and an important place of glucose and lipid metabolism, including digestion, absorption, transportation, catabolism, and anabolism, all of which are closely associated with the liver. Hepatic steatosis and serum triglyceride level increases are commonly observed after radiation [30, 40, 41].

Bakshi et al. [42] revealed that low-dose radiation could immediately inhibit the expression of pyruvate kinase isozymes (PKM), pyruvate dehydrogenase (PDH), dihydrolipoamide S-acetyltransferase (DLAT), aldolase A (ALDO-A), and carnitine acetyltransferase (CRAT), all of which are important enzymes for glucose metabolic regulation. Early on, in the process of lipid metabolism, peroxisomal acyl-coenzyme A oxidase 1 (ACOX1) showed radiation-induced downregulation, whereas ACOX2 was upregulated. In addition, they found late peroxisome proliferation-activated receptor (PPAR)α-mediated metabolic alterations and late increases in the levels of cytochrome P450 (CYP450) enzymes. In mice that received low-dose radiation, damage to the mitochondrial ultrastructure and lipid deposition in hepatocytes increased compared with the nonirradiated controls, and much more severe RILI was identified in ApoE −/− mice [43], indicating RILI has an apparent association with lipid metabolism disorder.

Another study [44] identified that the contents of several hepatic pentose cycle metabolites, including glucose-6-phosphate, mannose-6-phosphate, and mannose-1-phosphate, increased after liver radiation. Glucose-6-phosphate is involved in glycolysis, glycogen metabolism, and the oxidative limb of the pentose phosphate pathway (PPP). NADPH produced by PPP can participate in biosynthetic pathways, such as fatty acid synthesis, and suppress ROS via the reduction of glutathione. Moreover, hepatic malate and fumarate contents were found to be significantly decreased, indicating a decrease in TCA cycle function.

## Inflammation, depletion of injured cells, and fibrosis

**Fig. 2:**
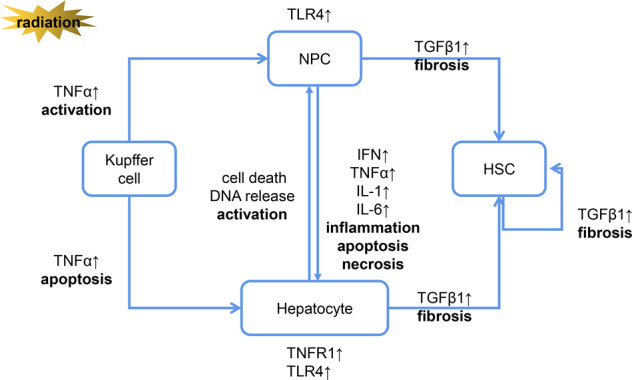
The activation of inflammatory response pathways in a variety of cells is involved in the development of RILI. Radiation directly induces hepatocyte injury through oxidative stress and inflammatory response and then aggravates liver injury by activating liver Kupffer cells and recruiting circulating immune cells to infiltrate and activate. In the late stage of RILI, NPCs and HSC are involved in the process of liver fibrosis mainly mediated by TGF-β1. NPC: nonparenchymal cell; HSC: hepatic stellate cells.

Inflammatory responses play a significant role in RILI (Fig. 2). Nuclear and mitochondrial DNA damage resulting from direct exposure to radiation or ROS leads to cell death via processes such as mitotic catastrophe, apoptosis, and primary and secondary necrosis [45] (Fig. 1). Necrosis can initiate the release of inflammatory cytokines [38], while apoptosis may cause the release of anti-inflammatory cytokines, including transforming growth factor (TGF)-β1 [6, 46, 47]. RILI ultimately translates into liver fibrosis due to the loss of hepatocytes and repair processes.

In a mouse model receiving stereotactic body radiation therapy, pathological changes, weight loss, and increases in serum hepatic enzymes were radiation dose-dependent in the range of 20 to 35 Gy [48], while also activating the apoptosis signal [33, 48] and autophagy signal [33]. Intralobular spotty necrosis and/or neutrophil infiltration around the vasculature were observed in the mouse livers in the 30 or 35 Gy groups [48].

Radiation can upregulate the expression of Toll-like receptor (TLR)4 in liver parenchymal and nonparenchymal cells (NPCs) and promote activation of the TLR4 signaling pathway [49, 50]. The activation of TLR4 signaling contributes to the secretion of inflammatory factors, such as tumor necrosis factor (TNF)-α, interleukin (IL)−1, IL-6, and cytokines [51], which subsequently elevates the infiltration of inflammatory cells, resulting in liver inflammation and injury [49, 52, 53].

NF-κB, which plays a predominant role in inflammation [54], can be activated by oxidative stress [55] and interacts with inflammation via a very complex mechanism. ROS can activate the NF-κB signaling pathway along with proinflammatory cytokines in RILI [6, 54, 56]. IL-6 and TNF-α secretion induced by NF-κB signaling can further aggravate inflammatory damage [54]. Radiation upregulates the expression of NF-κB-related genes (TRAF6, NIK, RELB, IKK, RELA) involved in both canonical and noncanonical NF-κB pathways [57] in hepatocytes and NPCs [56]. In addition, increased RelA(p65) expression [55], as well as nuclear translocation [31, 39], has been observed in RILI. Several studies have confirmed that anti-inflammatory and antioxidant agents can alleviate the development of RILI accompanied by decreased NF-κB expression [31, 55].

Genomic instability triggers the inflammatory response. Recent studies revealed that the cytosolic DNA sensing pathway has emerged as the major link between DNA damage and innate immunity [58, 59]. The cyclic GMP-AMP synthase (cGAS)–stimulator of interferon genes (STING) pathway connects DNA damage to inflammation [60]. After radiation, a large quantity of free double-stranded DNA (dsDNA) is released by injured hepatocytes. cGAS-STING signaling is rapidly activated by dsDNA in liver NPCs, causing interferon (IFN)-I production and release and concomitant hepatocyte damage [56]. Additionally, the activation of cGAS-STING can upregulate NF-κB (p50/p65) nuclear translocation and transcriptional activity [59, 61].

KCs are also involved in the development of RILI. After radiation exposure, TNF-α secretion increases in KCs, and the level of TNFR1 increases in hepatocytes [62]. Antisense oligonucleotide inhibition of TNF-α has been suggested to attenuate apoptosis in RILI [62]. In another study, the authors noted that GdCl3, a selective inhibitor of KCs, could reduce radiation-induced IL-1β, IL-6, and TNF-α production and ameliorate acute RILI [63]. GdCl3 pretreatment decreased the number of apoptotic hepatocytes and liver sinusoidal endothelial cells (LSECs) and also decreased hepatic steatosis [63]. Therefore, KC-derived TNF-α and the subsequent activation of TNFR1 in hepatocytes promote the development of RILI.

TGF-β1 is a cytokine that regulates the production, degradation, and accumulation of extracellular matrix (ECM) proteins. It plays a pivotal role in fibrosis that follows tissue damage in many vital organs and tissues, and its levels correlate with the degree of fibrosis [64]. TGF-β1 expression is significantly increased in the liver following radiation and the development of fibrosis [40, 41], and the extent of fibrosis correlates with the magnitude of this increase [65]. Certain inflammatory cells, hepatic stellate cells (HSCs), mesenchymal cells and epithelial cells may be involved in the intricate process of radiation‑induced liver fibrosis by acting as cellular sources of active TGF-β1 [39, 66, 67]. TGF-β1 can induce fibrosis via activation of both the canonical and noncanonical signaling pathways, which results in the activation of myofibroblasts, excessive production of ECM and inhibition of ECM degradation [64]. Excess ROS generated from radiation can disrupt the noncovalent bonds between latency-associated peptide (LAP) and TGF-β1; afterward, activated TGF-β1 results in the phosphorylation and activation of small mothers against decapentaplegic (SMAD) after binding to the receptor [7]. Then, the complex consisting of SMAD4 and phosphorylated SMAD2 and SMAD3 translocates to the nucleus to transcribe specific genes. SMAD3 can exert profibrotic functions in several ways [64]: (1) it can bind directly to gene promoters to induce transcription of profibrotic molecules, including α-smooth muscle actin (α-SMA), collagen I and tissue inhibitor of matrix metalloproteinases (TIMP), which results in myofibroblast activation and matrix deposition; (2) SMAD3 can induce transcription of profibrotic microRNA (miRNA) and long noncoding RNA (lncRNA) to inhibit the transcription of antifibrotic miRNAs; and (3) SMAD3 can increase the transcription of profibrotic molecules by influencing epigenetic modifications of DNA and histone proteins. The canonical pathway of TGF-β1-SMAD is referred to as the core axis that induces the differentiation of fibroblasts to myofibroblasts in several organs. It is crucial to the initiation and/or perpetuation of radiation-induced fibrosis [7, 66]. Additionally, ROS can regulate TGF-β1 signaling via noncanonical (SMAD-independent) mechanisms that are essential for normal profibrotic gene expression in many systems [68]. Furthermore, TGF-β1 can stimulate prolonged production of ROS in hepatocytes [69]. This positive feedback may aggravate the development and pathogenesis of late radiation-induced fibrosis in normal tissues. Inhibition of TGF-β signaling using soluble TGF-β type II receptor protein attenuates radiation-induced liver fibrosis in rats [65]. Hu et al. [70] reported that paeoniflorin treatment can attenuate radiation-induced hepatic fibrosis by inhibiting the TGF-β1-SMAD signaling pathway. Xiao et al. [68] declared that HSC activation, the central link of fibrosis, could be triggered by the TGF-β1-mediated PI3K/Akt signaling (noncanonical) pathway after radiation.

Wang et al. [40] found that activity of the Hedgehog (Hh) pathway increased in response to RILI and induced compensatory proliferation of liver progenitors and myofibroblastic hepatic stellate cells (MF-HSCs), thereby promoting liver fibrosis. Six weeks after a single dose of radiation, the RNA expression of ihh (a Hh ligand), smo (a Hh receptor), and gli2 (a Hh target gene) showed a great increase. The levels of liver triglycerides, TGF-β, α-SMA, and collagen α1 increased, whereas the level of bone morphogenetic protein (BMP)7 decreased. Ten weeks after single-dose radiation, RNA expression of shh (another Hh ligand), Smo, and Gli2 steadily increased with obvious liver fibrosis [40]. Similarly, in a fractionated radiation mouse model, the Hh pathway was upregulated in acute and chronic RILI with elevated hepatocyte apoptosis and fibrosis [71]. Moreover, a Hh inhibitor decreased liver Hh activity in irradiated mice and attenuated the proliferation of hepatic progenitors, liver injury, and fibrosis [40].

## Radiation and microRNAs

miRNAs play important roles in the regulation of diverse biological effects, such as cell proliferation, apoptosis, differentiation, and cell responses to environmental stimulation, including radiation [72, 73]. They exert their regulatory effects at the posttranscriptional level by binding to target genes via base pairing with the mRNA 3′ untranslated region (3′ UTR) to downregulate protein expression.

DNA damage caused by radiation can induce miRNA expression in an ATM kinase-dependent manner [74]. Activated ATM kinase induced by DSBs phosphorylates transcription factors, such as cAMP response element-binding protein (CREB) and p53, which are responsible for a large portion of miRNA expression by binding to the promoter region [74]. In addition, activated ATM kinase can posttranscriptionally regulate the biogenesis of many miRNAs through phosphorylation of breast cancer 1 (BRCA1) and KH-type splicing regulatory protein (KSRP), both of which are key components of both the Drosha and Dicer complexes [74]. Little is known about the regulatory mechanisms of ATM-independent miRNAs.

In an RILI mouse model, 48 differentially expressed miRNAs were identified through high-throughput deep sequencing technology and compared with mice that did not receive radiation [73]. Further analysis revealed that the predictive target genes of these miRNAs took part in an extensive range of biological effects, including transcription, modification, cell proliferation, and repair [73]. Additionally, the Kyoto Encyclopedia of Genes and Genomes (KEGG) pathways associated with radiation-induced differentially expressed miRNAs included “Pathways in cancer”, “TGF-β signaling”, “MAPK signaling”, “Focal adhesion”, “Apoptosis” and the “Wnt signaling pathway” [75].

Radiation increases miR-34a expression in the liver without a relationship between the expression level and radiation dose [73, 76]. miR-34a, a p53 transcriptional target, can induce p53-mediated apoptosis, cell cycle arrest in the G1 phase and senescence [76]. In addition, miR-34a can activate p53 by directly inhibiting SIRT1 and HDM4 [77, 78], a potent negative regulator of p53, and indirectly inhibiting HDM2 [77]. Therefore, miR-34a can increase p53 protein levels and stability, creating a positive feedback loop that acts on p53 [78]. In addition, miR-34a regulates a variety of target genes involved in the cell cycle, cell proliferation, senescence, migration, and invasion [77]. For example, miR-34a can regulate cell apoptosis by influencing the phosphorylated key protein levels in mitogen-activated protein kinase (MAPK) signaling through mediating MAP3K9 [79] and MAP3K10 [80] expression. In addition, miR-34a can induce cell cycle arrest, especially during cell proliferation and senescence, by interacting with its target genes N-MYC, CCND1, CCNE2, CDK4, CDK6, and MET [81–83]. It has been confirmed that miR-34a can induce cell senescence through four main methods: the p53/miR-34a/SIRT1 axis, the miR-34a/E2F/RB axis, the miR-34a/HBP1/RAS axis and the miR-34a/MAPK/p16 axis [77]. In normal tissue receiving radiation exposure, miR-34a can aggravate tissue injury by promoting DNA damage, cytokine production, and cell senescence or suppressing cell cycle progression and antioxidant molecules [76]. Chen et al. [84] reported that overexpression of miR-34a-5p directly reduced the expression of Krüppel-like factor 4 (KLF4) and induced hepatocyte apoptosis after radiation exposure.

In mice fed a high-fat diet, the expression of miR-466e-5p was upregulated, while a miR-466e inhibitor can counteract free fatty acid-triggered radiation sensitization [75].

miR-146a-5p is a key regulator of lipopolysaccharide (LPS)/TLR4 signaling. Chen et al. [50] found that miR-146a-5p was upregulated in HSCs after radiation. Overexpression of miR-146a-5p, which attenuates hepatocyte apoptosis and liver fibrosis, can inhibit cell proliferation, proinflammatory cytokine secretion, and cell activation in radiated HSCs by downregulating the expression of TLR4 [50].

Fu et al. [85] demonstrated that miR-495 was downregulated after radiation. Overexpression of miR-495 could alleviate RILI by targeting the transcription factor 1 (Sp1)/endothelial nitric oxide synthase (eNOS) pathway. Consequently, nitric oxide (NO) and its downstream product TGF-β1 were inhibited after radiation-induced injury.

Extracellular vesicles (EVs), such as exosomes and microvesicles, are cell-derived membranous surrounding vesicles [86]. Most cells can release EVs for intercellular communication [87]. The contents of EVs comprise proteins, messenger RNAs (mRNAs), miRNAs and DNA derived from the cell of origin; therefore, EVs are cell type- and cell condition-specific. miRNAs in EVs can be delivered to bystander cells to exert regulatory functions [87]. For example, senescent cells can release senescence-associated miRNAs shuttled by EVs to spread prosenescence signals [88, 89]. Radiation can promote EV release in a dose-dependent manner [90]. However, studies focusing on radiation-induced changes in EV content are limited. miR-21 and miR-34c transferred by EVs from radiated cells can mediate bystander effects in nonradiated cells [91, 92]. miRNAs in mesenchymal stem cell (MSC)-derived EVs also play an important role in radiation-induced injury. miR-214-3p transferred to MSC-derived EVs attenuates radiation-induced injury of endothelial cells in the lung by inhibiting the ATM/p53/p21 signaling pathway and SASP development [93]. miR-214 in human neural stem cell-derived EVs ameliorates radiation-induced brain injury [94]. However, data on miRNA-mediated intercellular communication and therapy during RILI are lacking.

Overall, miRNAs induced by radiation can participate in the development of RILI by binding to diverse target genes and exerting complex effects (Table 3). In addition, miRNAs can also be transferred from donor cells to bystander cells by EVs to promote RILI development. In addition, specific miRNAs in MSC-derived EVs may provide a new avenue to minimize RILI. Further investigations should be conducted to explore the potential functions of miRNAs.

**Table 3:** MiRNA involved in RILI.

miRNA	Mechanism	Biological effect	Response	Ref
miR-34a	Increase p53 protein levels and stability, and create a positive feedback loop acting on p53.	Induce p53 mediated apoptosis, cell cycle arrest in the G1 phase, and senescence, senescence, migration, and invasion.	Increase	[45, 48, 49]
miR-34a-5p	Reduces the mRNA and protein levels of KLF4.	Negatively regulate KLF4 expression and promote apoptosis.	Increase	[50]
miR-466e-5p	Might involve lipid metabolism.	Modulate radiation responses in diet-induced obesity.	/	[47]
miR-146a-5p	Inhibit the TLR4 signaling pathway.	Attenuate radiation-induced hepatic stellate cell activation and hepatocyte apoptosis	Increase	[35]
miR-495	Indirectly downregulate eNOS and NO production via targeting Sp1 and inhibit NO and its downstream product TGF -β1.	Alleviate cell injury.	Decrease	[51]

## Conclusion and future directions

The present study aimed to profile the overall mechanism of RILI and the effects of radiation on hepatocyte senescence. RILI is a major complication of radiotherapy for the treatment of liver cancer and other upper abdominal malignant tumors [11, 95]. RILI can increase the risk of liver dysfunction and liver failure and seriously affect subsequent treatments and prognosis [11]. Lacking pharmacological therapies, the management of RILI remains a major problem in clinical practice.

DNA damage caused by radiation is the main reason for cell senescence in RILI [4, 20]. Additionally, the death of numerous hepatocytes caused by radiation may increase the effects of replicative aging. Senescence can deteriorate liver function, cell viability and tissue regeneration under pathological conditions. Hepatic senescence without proliferative ability causes the liver to be much more susceptible to harmful factors and may contribute to the deterioration of RILI. SASP secreted from senescent cells can lead to changes in tissue homeostasis and the microenvironment. Senescent cell elimination has been proven to be beneficial to radiation-induced injury in several studies. Although evidence on RILI is still lacking, treatment with senolytic agents is a very promising method.

After radiation exposure, the oxidative and antioxidant balance in the liver is impaired [6, 15, 28]. Oxidative stress results in structural changes and the dysfunction of proteins, lipids and nucleic acids and alters cell survival and metabolism. Interestingly, the effects of ROS are controversial. Various antioxidants have been confirmed to alleviate the progression of RILI by acting as ROS scavengers [28, 55, 96, 97]. Moreover, glibenclamide activates Akt-NF-κB signaling by upregulating cellular ROS, benefiting hepatocyte survival [39]. ROS form as a natural byproduct of normal oxygen metabolism, participating in cell signal transduction and homeostasis under physiological conditions. Therefore, compared with simply eliminating ROS, readdressing the balance of the oxidation/reduction system is the dominant direction in RILI.

Glucose and lipid metabolism disorders are observed even after low-dose radiation, and the effects will last a long time. Additionally, livers suffering from lipid metabolism disorder are more sensitive to RILI [43]. Attention needs to be paid to basic liver conditions when receiving abdominal radiotherapy or radiation exposure.

Hepatocyte necrosis and apoptosis occur after high-dose radiation, which activates the inflammatory response in the liver via numerous pathways [41, 51, 54]. The interaction between injured hepatocytes and liver NPCs promotes the process of inflammation and liver fibrosis through the activation of several critical pathways [56, 63]. Interventions aimed at the TNF-α, NF-κB, TGF-β, and Hh pathways can alleviate RILI. Similarly, inflammatory signal activation has two sides during the progression of RILI. On the one hand, it can aggravate liver injury in the early stage of RILI. On the other hand, it is necessary for organ proliferation and repair after severe damage [98]. You et al. [99] confirmed that the combined absence of KCs and infiltrating macrophages resulted in a marked delay in liver repair after acetaminophen-induced liver injury.

Radiation-induced miRNAs participate in preventing or promoting RILI via interactions with their target genes [73]. In addition to intracellular regulatory effects, miRNAs delivered by EVs, important mediums of cellular communication, may play an important role in nonradiative cell injury during RILI. miRNAs are widely involved in the regulation of oxidative stress, inflammation and aging. The regulatory role of miRNAs on radiosensitivity allows them to have the potential to be useful in clinical radiotherapy.

RILI is a multistep, dynamic process that involves a complicated network in which oxidative stress, inflammation, cell death, fibrosis, miRNAs and senescence interact via the regulation of multiple pathways. Restoring cellular homeostasis is critical for its treatment. The mechanism underlying RILI is not yet fully understood, highlighting the significance of continued research to clarify the role of different pathways in various liver cells. Future research on RILI is necessary to develop novel therapeutic interventions.

## Data Availability

Data sharing is not applicable to this article as no datasets were generated or analyzed during the current study.
